# Association of air pollution and 1-year clinical outcomes of patients with acute myocardial infarction

**DOI:** 10.1371/journal.pone.0272328

**Published:** 2022-08-01

**Authors:** Se Yeon Choi, Seung-Woon Rha, Jinah Cha, Jae Kyeong Byun, Byoung Geol Choi, Myung ho Jeong, on behalf of The Korea Acute Myocardial Infraction Registry study group

**Affiliations:** 1 Cardiovascular Research Institution, Korea University College of Medicine, Seoul, Korea; 2 Cardiovascular Center, Korea University Guro Hospital, Seoul, Korea; 3 BK21 Graduate Program, Department of Biomedical Sciences, Korea University College of Medicine, Seoul, Korea; 4 Division of Cardiology, Department of Medicine, Chonnam National University Hospital, Gwangju, Korea; Baylor Scott and White, Texas A&M College of Medicine, UNITED STATES

## Abstract

**Background:**

Exposure to air pollution (AP) is an important environmental risk factor for increased risk of cardiovascular morbidity and triggering acute myocardial infarction (AMI). However, there are limited data regarding the clinical impact of AP on long-term major clinical outcomes of AMI patients. This study aimed to evaluate the clinical effects of ambient AP concentration on short-term and 1-year clinical outcomes of AMI patients.

**Methods:**

A total of 46,263 eligible patients were enrolled in the Korea Acute Myocardial Infarction (KAMIR) and KAMIR-National Institutes of Health (NIH) registry from January 2006 to December 2015. We performed Cox proportional hazard regression to assess the risk of all-cause death and any-revascularization according to the annual average concentration of AP during one-year follow-up period.

**Results:**

The assessment of the annual average of air pollutants before symptom date and all-cause death up to 30 days showed the hazard ratio (HR) of SO_2_ per 1 part per billion (ppb) increase was 1.084 (95% confidence interval [CI]: 1.016–1.157), and particulate matter with diameter of 10 microns or less (PM_10_) per 1 μg/m^3^ increase was 1.011 (95% CI: 1.002–1.021). The results of the 30-day and one-year all-cause death showed a similar trend. For SO_2_, the HR per 1 ppb increase was 1.084 (95% CI: 1.003–1.172), and the HR of PM_10_ was 1.021 (95% CI: 1.009–1.033) per 1 μg/m^3^ increase. We observed that SO_2_, CO, and PM_10_ were associated with an increased risk of incidence for any-revascularization up to one-year.

**Conclusion:**

In some air pollutants, a higher AP concentration was an environmental risk factor for poor prognosis in AMI patients up to 1 year. AMI patients and high-risk individuals need a strategy to reduce or prevent exposure to high AP concentrations.

## Introduction

Exposure to ambient air pollution (AP) is an inevitable circumstance for the population, and it is an important environmental risk factor for adverse health effects. AP concentrations in most Asian countries exceed the air quality guidelines of the World Health Organization, and concentrations in low- and middle-income countries are relatively high and increasing [1]. The population living in these countries is estimated to have a large burden of adverse health effects due to exposure to AP, and is expected to gradually increase depending on AP concentration. The impact of AP on human health is known to cause 4.2 million premature deaths worldwide per year, and is related to not only respiratory diseases but many acute and chronic diseases [2–5]. In addition, short- and long-term exposure to AP is associated with cardiovascular diseases such as the increased risk of cardiovascular morbidity, triggering acute myocardial infarction (AMI), and even increased cardiovascular death via pulmonary and systemic inflammation [6–11].

The majority of AP-related studies that have been conducted focused on the onset and relevance of disease based on the concentration of exposure to AP in the general population. There are limited data on the clinical aspects of patients with specific diseases. Our previous study investigated the association between short-term clinical outcomes and AP exposure in AMI patients. We found that AP exposure within 24 h before AMI admission was associated with mortality and cardiovascular events [12]. This study was conducted via an extended period of clinical follow-up and AP concentration data as a serial study followed by our previous study and aimed to evaluate the clinical effects of ambient AP concentration in AMI patients using nationwide prospective multicenter registry data.

## Materials and methods

### Study population

**Fig 1:**
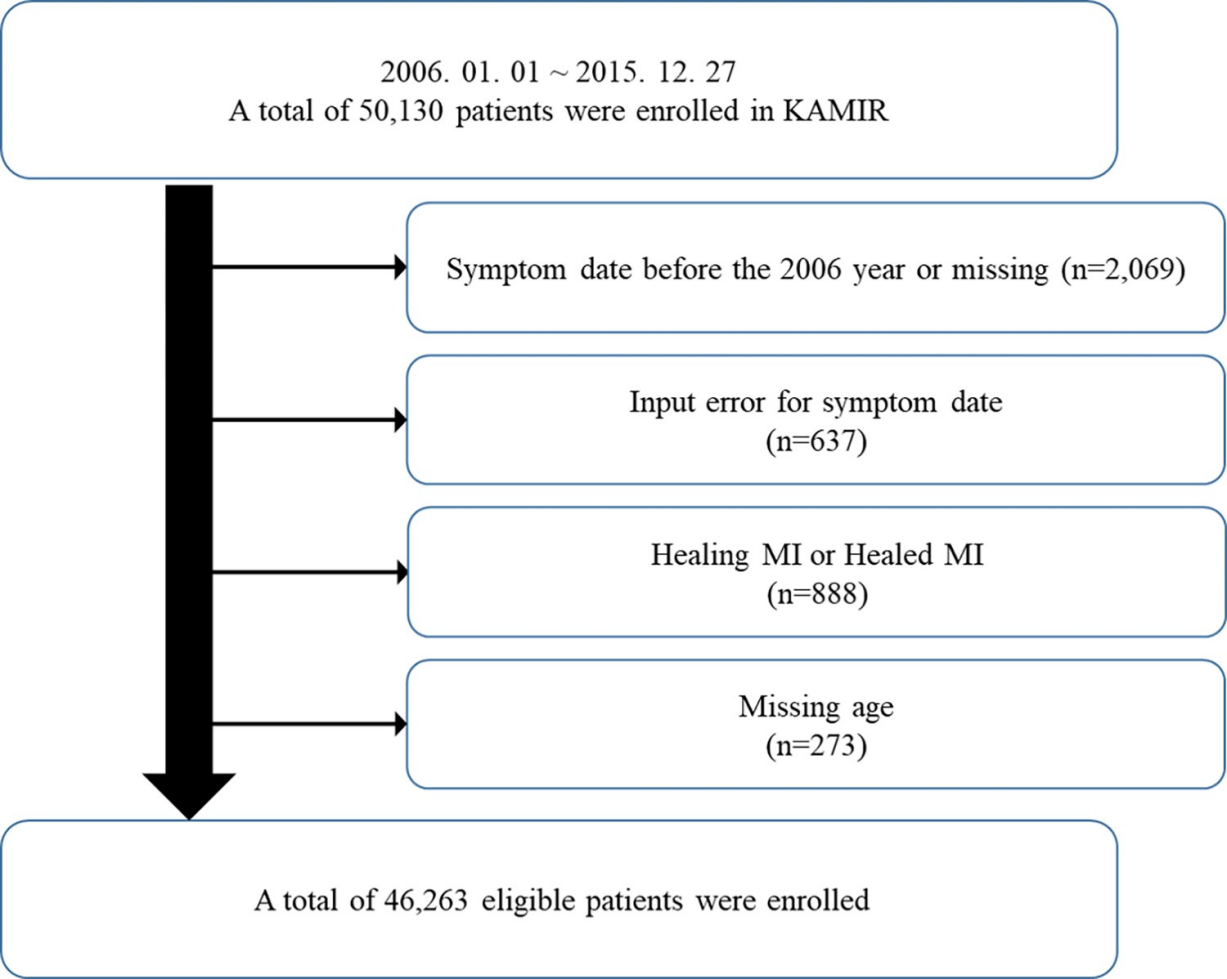
Flow chart of study population.

The data was collected from the Korea Acute Myocardial Infarction (KAMIR) and KAMIR-National Institutes of Health (KAMIR-NIH). The KAMIR study design has been introduced in previous studies [12, 13]. KAMIR and KAMIR-NIH are a nationwide prospective multicenter registration study series that aims to establish treatment guidelines and derive risk factors through various clinical characteristics and follow-up of Korean AMI patients from October 2005. A flowchart of the study is shown in Fig 1. A total of 50,130 AMI patients were enrolled in the KAMIR and the KAMIR-NIH from January 2006 to December 2015. Exclusion criteria were (1) symptom data before the year 2006 or missing year, (2) input error for symptom date, (3) healing MI or healed MI, and (4) missing important demographic factors such as age and sex. A total of, 46,263 eligible patients were included in the study. The study protocol was approved by the Korea University Guro Hospital Institutional Review Board (#2011GR0481J). All patients received information of participation in this study and provided written informed consent.

### AP measurement

Air pollutant measurement data, which are available on the Air Korea website (http://www.airkorea.or.kr) operated by the Korean Ministry of Environment has been provided. The monitoring station measured PM_2.5_ by mass concentration, PM_10_ by the β-ray absorption method, CO by the non-dispersive infrared method, SO_2_ by pulse ultraviolet fluorescence method, NO_2_ by chemiluminescence method, and O_3_ by ultraviolet photometric method. The measurement of PM_2.5_, began in January 2015, while the other pollutants began in 2001.

We collected hourly concentrations of AP data from 329 monitoring stations nationwide and then transformed them into the daily average value. An individual’s exposure concentration to air pollutants was measured by matching each monitoring station with 68 hospitals registered in KAMIR in the order of the closest straight line. Since addresses of patients were unavailable from the multicenter registry, the monitoring station was selected based on the admitted hospital. Because AMI is an emergent event, patients would have been admitted to the nearest hospital, which was the closest to their residency or workplace at the time of disease onset. When a missing value occurred owing to problems such as breakdown and connection error with the monitoring station, the value of the next nearest monitoring station was inputted. The reference date was based on the onset of the symptom date defined the first time of MI related symptoms such as chest pain or dyspnea, and the annual average value of air pollutants before and after the symptom day was calculated.

### Study definitions and study endpoints

AMI were defined as the presence of clinical symptoms, changes in electrocardiogram (EKG) indicating new ischemic signs, and elevation of cardiac enzymes by at least one value above the upper limit of the reference range until 7 days after symptom onset. Individual cardiovascular risk factors such as hypertension, dyslipidemia, diabetes mellitus (DM), prior cardiovascular disease, heart failure (HF), prior cerebrovascular disease, and smoking history were based on patient self-reports. The study endpoints were the cumulative incidence of all-cause death and any-revascularization during a one-year clinical follow-up period. All-cause death was defined as the incidence of death from cardiac or non-cardiac origin. Any-revascularization was defined as revascularization of the target vessel or non-target vessel revascularization.

### Statistical analysis

All statistical analyses were conducted in R version 4.0.2 (R Core Team, 2020; R: Language and Environment for Statistical Computing; R Foundation for Statistical Computing, Vienna, Austria. URL: https://www.R-project.org/). Continuous variables were described as mean ± standard deviation, and categorical data were expressed as percentages.

We performed Cox proportional hazard regression and stratified the hospitals to assess the hazard ratio (HR) of each air pollutant with a 95% confidence interval (CI) in terms of the clinical outcomes, and to account for the hospital and regional effects such as accessibility and treatment plans. Using a multivariable model, we adjusted all available variables that could be potentially relevant factors: age, sex, body mass index, smoking status, ST-segment elevation MI (STEMI), hypertension, DM, dyslipidemia, stroke, HF, previous ischemic heart disease, percutaneous coronary intervention (PCI), multi-vessel disease, left main disease, cardiopulmonary resuscitation (CPR), left ventricular ejection fraction (LVEF), and symptom date. Statistical significance was defined as a p-value of < 0.05.

## Results

Baseline clinical and angiographic characteristics are shown in Table 1. The mean age of the study population was 63.8 years old, 72.1% were male, 54.3% were diagnosed with STEMI, and 3.9% of them received CPR before hospitalization. PCI was the priority AMI treatment in 86.7% of patients, and 46.8% showed multi-vessel disease on coronary angiography.

**Table 1:** Baseline and clinical characteristics.

Variables	Total (n = 46,263)
Age, year	63.8 ± 12.8
Sex (Male)	33,336 (72.1%)
Body mass index, kg/m^2^	24.0 ± 3.3
ST-segment elevation MI	25,101 (54.3%)
Cardiopulmonary resuscitation	1,806 (3.9%)
Left ventricular ejection fraction, %	52.0 ± 11.9
Previous Ischemic heart disease	7,155 (15.5%)
Previous PCI	3,431 (7.4%)
Previous MI	2,233 (4.8%)
Previous CABG	377 (0.8%)
Previous angina	2,859 (6.2%)
Hypertension	23,096 (49.9%)
Diabetes mellitus	12,764 (27.6%)
Dyslipidemia	5,143 (11.1%)
Cerebrovascular disease	3,077 (6.7%)
Heart failure	864 (1.9%)
Smoking history	26,614 (57.5%)
Current smoker	19,026 (41.1%)
Family history of heart disease	3,315 (7.2%)
Initial treatment of MI	
PCI	40,100 (86.7%)
CABG	937 (2.0%)
Thrombolysis	991 (2.1%)
Multi-vessel disease	21,664 (46.8%)
Left main disease	1,670 (3.6%)
Infarct-related artery	
Left main	889 (1.9%)
Left anterior descending artery	19,075 (41.2%)
Left circumflex artery	6,892 (14.9%)
Right coronary artery	13,557 (29.3%)
**One-year clinical outcomes**	
All-cause death	3,469 (7.5%)
Cardiac death	2,981 (6.4%)
Any-revascularization	1,776 (3.8%)

MI, myocardial infarction; PCI, percutaneous coronary intervention; CABG, coronary artery bypass graft.

**Fig 2:**
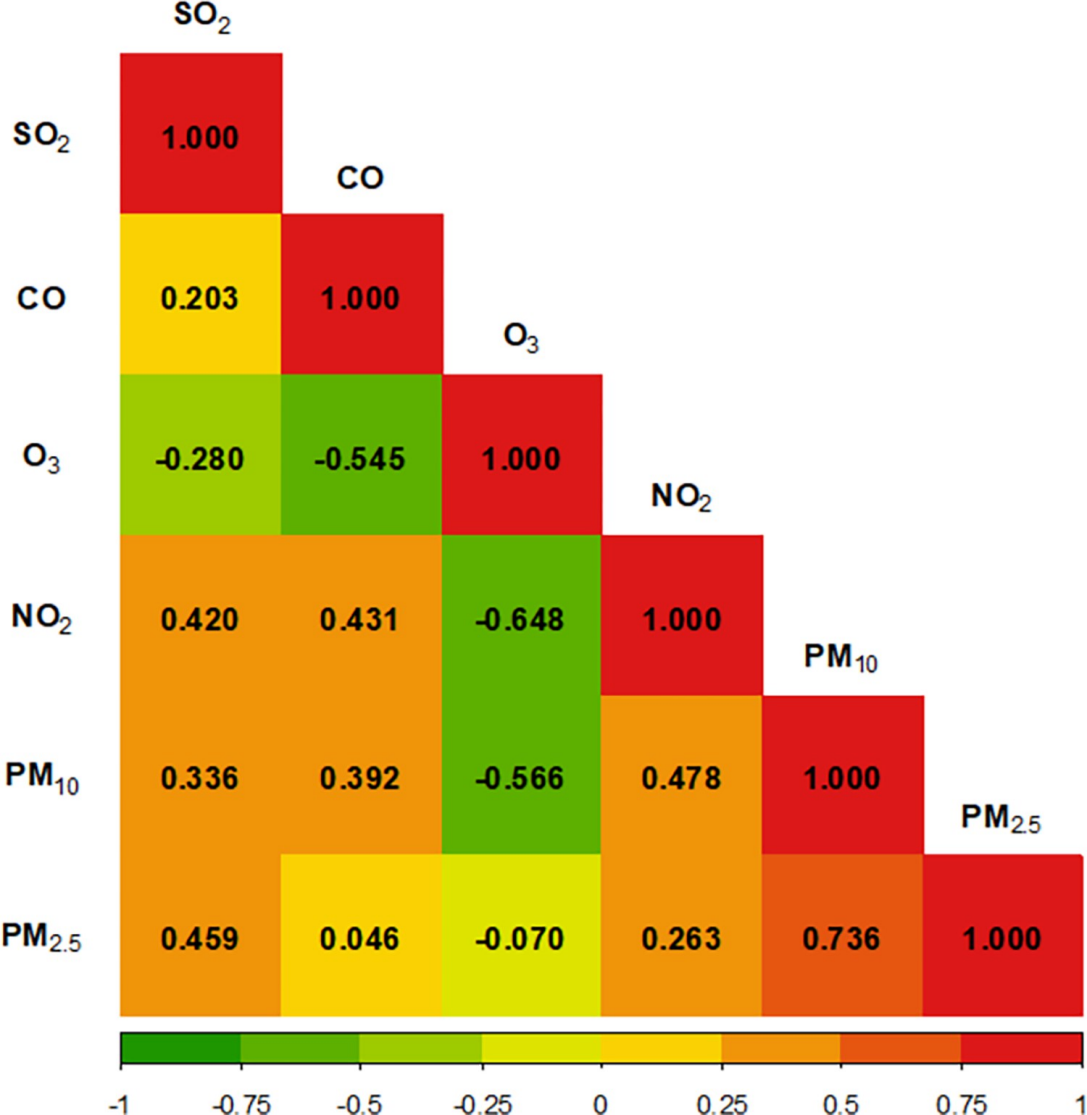
Spearman correlation coefficients for annual average concentrations of air pollutants.

During the follow-up period of AMI patients, the median value of annual average concentrations was 0.049 part per million (ppm) for SO_2_, 0.5882 ppm for CO, 0.0216 ppm for O_3_, 0.0253 ppm for NO_2_, 48.88 μg/m^3^ for PM_10_, and 24.01 μg/m^3^ for PM_2.5_ (Table 2). In the Spearman rank correlation analysis using average annual concentrations after symptom date, most air pollutants showed a positive correlation (r = 0.0462 to 0.736); however, O_3_ and other air pollutants showed a negative correlation (r = -0.0705 to -0.648, Fig 2).

**Table 2:** Distribution of annual average of air pollution concentration after symptom date.

	SO_2_, ppm	CO, ppm	O_3_, ppm	NO_2_, ppm	PM_10_, ㎍/㎥	PM_2.5_, ㎍/㎥
0–20% (Q1)	0.0015–0.0039	0.2426–0.4808	0.0067–0.0172	0.0070–0.0209	29.28–41.96	19.25–20.49
20–40% (Q2)	0.0039–0.0045	0.4808–0.5588	0.0172–0.0207	0.0209–0.0234	41.96–46.67	20.49–22.25
40–60% (Q3)	0.0045–0.0052	0.5588–0.6220	0.0207–0.0228	0.0234–0.0282	46.67–50.89	22.25–26.71
60–80% (Q4)	0.0052–0.0063	0.6220–0.7270	0.0228–0.0262	0.0282–0.0355	50.89–57.82	26.71–29.50
80–100% (Q5)	0.0063–0.0111	0.7270–1.4991	0.0262–0.0436	0.0355–0.0766	57.82–97.22	29.50–41.54
Median	0.0049	0.5882	0.0216	0.0253	48.88	24.01
Mean	0.0051	0.6002	0.0215	0.0280	50.26	25.56
Interquartile range (IQR)	0.0019	0.1988	0.007	0.0123	12.15	7.24

The risk of all-cause death and any-revascularization at up to one-year regarding the average annual AP concentrations after symptom date are shown in Table 3. Most air pollutants were not associated with the risk of all-cause death at up to one-year, except for CO, which was observed to decrease risk (HR per 1 part per billion [ppb] increase: 0.938, 95% CI: 0.884–0.994; HR for Q2: 1.213, 95% CI: 1.010–1.457; HR for Q5: 0.728, 95% CI: 0.568–0.933). We observed that SO_2_, CO, and PM_10_ were associated with an increased risk of incidence of any-revascularization at up to one-year. The HR of SO_2_ was 1.113 (95% CI: 1.054–1.175) per 1 ppb increase, wherein Q4 (HR: 1.459, 95% CI: 1.187–1.793) and Q5 (HR: 1.605, 95% CI: 1.273–2.025) were different from those of the first quintile. The HR of CO was 1.136 per 0.1 ppm increase (95% CI: 1.067–1.210), with a difference at Q5 (HR: 1.622, 95% CI: 1.244–2.114). The HR of PM_10_ was 1.020 per 1 μg/m^3^ (95% CI: 1.012–1.028), and with a difference from Q2 to Q5 (HR for Q2: 1.629, 95% CI: 1.343–1.976; HR for Q3: 1.649, 95% CI: 1.374–1.980; HR for Q4: 1.844, 95% CI: 1.477–2.303; HR for Q5: 2.000, 95% CI: 1.552–2.578).

**Table 3:** Adjusted hazard ratio and 95% confidence interval of the incidence of all-cause death and any-revascularization regarding annual average concentration of each air pollutants after symptom date.

	All-cause death		Any-revascularization	
	Hazard Ratio (95% CI)	P-value	Hazard Ratio (95% CI)	P-value
SO_2_, ppb	0.986 (0.937–1.038)	0.586	1.113 (1.054–1.175)	<0.001
SO_2_ Q_1_	**Reference**		**Reference**	
SO_2_ Q_2_	1.091 (0.930–1.280)	0.287	1.035 (0.869–1.234)	0.698
SO_2_ Q_3_	1.052 (0.896–1.234)	0.539	1.181 (0.993–1.403)	0.060
SO_2_ Q_4_	1.308 (1.085–1.577)	0.005	1.459 (1.187–1.793)	<0.001
SO_2_ Q_5_	0.951 (0.759–1.192)	0.662	1.605 (1.273–2.025)	<0.001
CO, 0.1 ppm	0.938 (0.884–0.994)	0.032	1.136 (1.067–1.210)	<0.001
CO Q_1_	**Reference**		**Reference**	
CO Q_2_	1.213 (1.010–1.457)	0.039	1.088 (0.875–1.353)	0.449
CO Q_3_	1.086 (0.891–1.324)	0.415	0.921 (0.727–1.166)	0.491
CO Q_4_	0.938 (0.756–1.164)	0.561	1.272 (0.994–1.627)	0.056
CO Q_5_	0.728 (0.568–0.933)	0.012	1.622 (1.244–2.114)	<0.001
O_3_, ppb	0.987 (0.967–1.009)	0.242	0.984 (0.963–1.006)	0.144
O_3_ Q_1_	**Reference**		**Reference**	
O_3_ Q_2_	0.557 (0.450–0.690)	<0.001	0.965 (0.766–1.216)	0.763
O_3_ Q_3_	0.693 (0.535–0.896)	0.005	1.159 (0.892–1.506)	0.270
O_3_ Q_4_	0.716 (0.544–0.942)	0.017	0.873 (0.652–1.170)	0.364
O_3_ Q_5_	0.712 (0.526–0.963)	0.028	0.921 (0.667–1.270)	0.614
NO_2_, ppb	1.001 (0.989–1.014)	0.867	1.007 (0.995–1.018)	0.266
NO_2_ Q_1_	**Reference**		**Reference**	
NO_2_ Q_2_	1.009 (0.839–1.214)	0.925	0.989 (0.806–1.213)	0.915
NO_2_ Q_3_	1.012 (0.821–1.249)	0.910	1.307 (1.047–1.631)	0.018
NO_2_ Q_4_	0.831 (0.628–1.101)	0.197	0.914 (0.683–1.223)	0.545
NO_2_ Q_5_	1.070 (0.768–1.492)	0.688	1.215 (0.874–1.691)	0.247
PM_10_, ㎍/㎥	0.993 (0.986–1.001)	0.100	1.020 (1.012–1.028)	<0.001
PM_10_ Q_1_	**Reference**		**Reference**	
PM_10_ Q_2_	1.062 (0.897–1.257)	0.484	1.629 (1.343–1.976)	<0.001
PM_10_ Q_3_	0.861 (0.725–1.023)	0.088	1.649 (1.374–1.980)	<0.001
PM_10_ Q_4_	0.695 (0.556–0.868)	0.001	1.844 (1.477–2.303)	<0.001
PM_10_ Q_5_	0.794 (0.614–1.027)	0.079	2.000 (1.552–2.578)	<0.001
PM_2.5_, ㎍/㎥	1.119 (0.781–1.603)	0.542	0.989 (0.780–1.255)	0.929
PM_2.5_ Q_1_	**Reference**		**Reference**	
PM_2.5_ Q_2_	0.766 (0.381–1.539)	0.454	0.736 (0.330–1.645)	0.456
PM_2.5_ Q_3_	0.978 (0.182–5.251)	0.979	0.416 (0.105–1.639)	0.210
PM_2.5_ Q_4_	1.338 (0.110–16.288)	0.820	1.569 (0.235–10.485)	0.642
PM_2.5_ Q_5_	0.963 (0.034–27.354)	0.982	-	

Adjusted by Age, Sex, Body mass index, Smoker, ST-segment elevation myocardial infarction, Hypertension, Diabetes mellitus, Dyslipidemia, Stroke, Heart failure, Previous ischemic heart disease, Percutaneous coronary intervention, Multi-vessel disease, Left Main Disease, Cardiopulmonary resuscitation, Left ventricular ejection fraction and symptom date

CI; Confidence interval, ppm; part per million, ppb; part per billion

**Fig 3:**
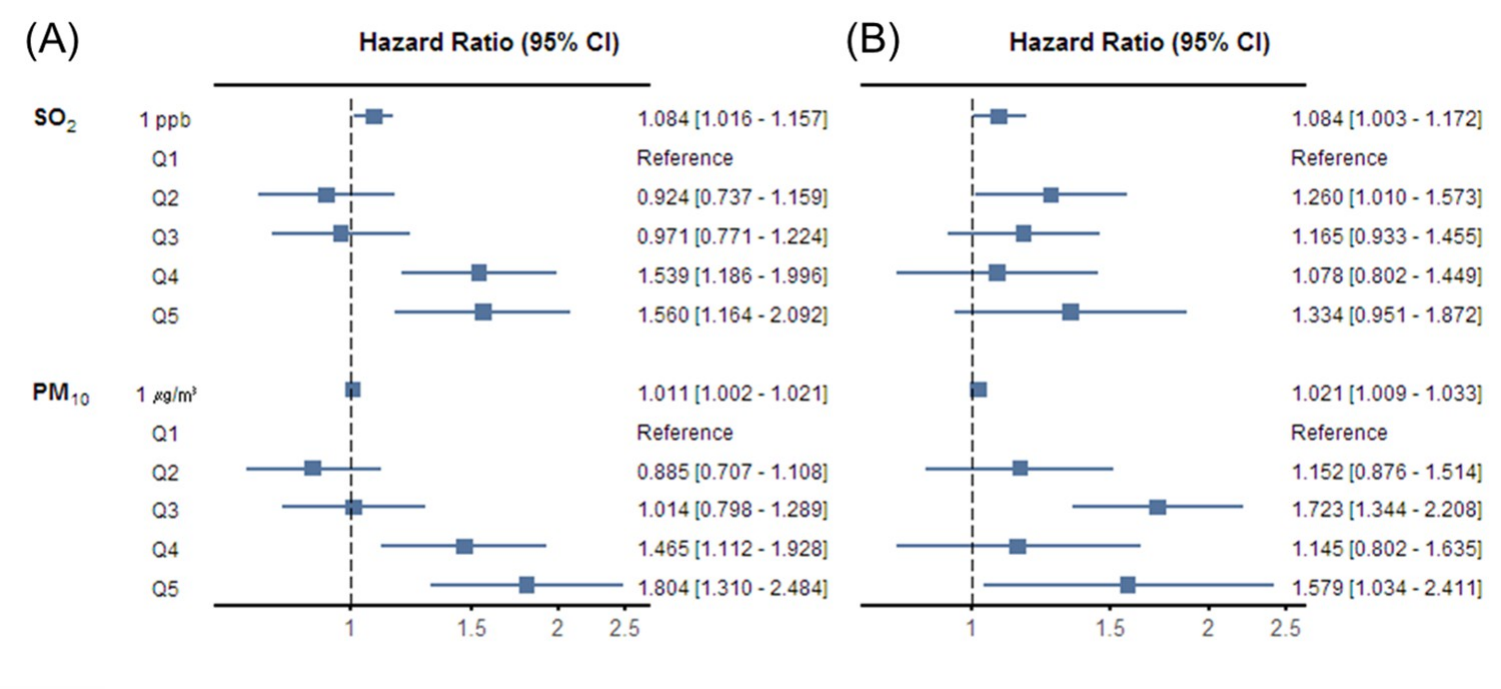
Adjusted hazard ratio and 95% confidence interval of all-cause death regarding SO_2_ and PM_10_. (A) Association between annual average concentration before symptom date and all-cause death up to 30 days, and (B) association between annual average concentration after symptom date and all-cause death after 30 days to one-year. CI; confidence interval.

In this study, since most all-cause deaths occurred within 30 days, we performed a risk assessment by corresponding the annual average before symptom date with that observed at up to 30 days and annual average after symptom date with that of up to one-year after 30 days. The cumulative incidence of all-cause deaths at up to 30 days and up to one-year after 30 days were 5.4% and 2.3%, respectively (S1 Fig). Based on the symptom date, the annual average concentration range of ambient air pollutants for AMI patients before and after treatment were similar (S1 Table). The assessment of the annual average of air pollutants before symptom date and all-cause death up to 30 days showed that SO_2_ and PM_10_ were related to having increased risk. The HR of SO_2_ per 1 ppb increase was 1.084 (95% CI: 1.016–1.157), with a difference from Q4 (HR: 1.539, 95% CI: 1.186–1.996) and Q5 (HR: 1.560, 95% CI: 1.164–2.092), and PM_10_ per 1 μg/m^3^ increase was 1.011 (95% CI: 1.002–1.021), with a difference from Q4 (HR: 1.465, 95% CI: 1.112–1.928) and Q5 (HR: 1.804, 95% CI: 1.310–2.484). The results after the 30-day up to one-year all-cause death showed some difference but it seemed that the risk was associated with a high concentration of AP. For SO_2_, the HR per 1 ppb increase was 1.084 (95% CI: 1.003–1.172), with a Q2 difference only (HR: 1.260, 95% CI: 1.010–1.573). PM_10_ showed an increase in HR per 1 μg/m^3^ increase, Q3 and Q5 by 1.021 (95% CI: 1.009–1.033), 1.723 (95% CI: 1.344–2.208), and 1.579 (95% CI: 1.034–2.411), respectively (Fig 3, S2 and S3 Tables).

We performed a subgroup analysis of the annual average of air pollutants before symptom date and all-cause death up to 30 days using continuous value. Most subgroups had similar effects by each air pollutant except that younger age (< 65 years) was increased risk per 1 ppb SO_2_ (HR: 1.179, 95% CI: 1.048–1.326, P interaction: 0.004) and NO_2_ (HR:1.040, 95% CI: 1.014–1.066, P interaction: 0.003) increase (S2–S6 Figs).

## Discussion

The present study provides strong evidence of an association between ambient AP exposure and 1-year major clinical outcomes in patients with AMI. The results imply that the risk of mortality and recurrent cardiovascular disease may be affected by high AP concentration during the periods not only after but also before hospitalization. The strength of this study was that it focused on the clinical aspects more than prior studies had and measured the effect of AP exposure over a certain time period on each individual.

Several studies have reported that short- and long-term exposure to AP is associated with mortality in cardiovascular disease patients [11, 12, 14–18]. Long-term exposure to PM_10_ from the year of death or end of the follow-up to 3 years prior was associated with an increase in the HR to 1.3 (95% CI: 1.2–1.5) per 10 μg/m^3^ in MI patients [16]. Another cohort study in acute coronary syndrome patients reported that increase in NO_2_, NO_x_, PM_10_, and PM_2.5_ during follow-up periods were associated with increased all-cause mortality in the adjusted model, including demographic and clinical factors; however, the mutually adjusted model with added pollutant and income, showed that only PM_2.5_ was related to increased mortality [17]. In our previous study using the same registry, short-term exposure to NO_2_, SO_2_, and CO was related to increased mortality during the 30-day follow-up period [12]. This study was an extended analysis of exposure and follow-up duration, and the results demonstrated that SO_2_ and PM_10_ were associated with an increased risk of all-cause death. The results of this study suggest that changes in the concentrations of air pollutants may affect clinical outcomes. There seems to be no association with mortality during the entire follow-up period for one year. This result showed that most of the all-cause death occurred before 30 days, it may not reflect the annual average concentration of AP after AMI. Therefore, it will be necessary to estimate the association according to different time periods. In the case of all-cause death, we assessed the effect of the short-term outcome based on the concentration of AP of the symptom date and the effect of the long-term outcome based after the symptom date. It may explain more properly the association between all-cause death and AP according to time periods. As a result, the short-term results showed an association with SO_2_ and PM_10_ before AMI onset. The results of extended periods of exposure after the 30-day follow-up showed an association between mortality and AP concentrations during the follow-up periods. Therefore, even if the AP concentration before the onset of AMI was high, a decrease in the concentration after onset was expected to lead to a reduced risk of all-cause death.

Another finding of the study was that a high concentration of SO_2_, CO, and PM_10_ after the onset of AMI was closely related to a higher incidence of hospitalization due to revascularization. In particular, PM_10_, compared with the first quintile, showed that a difference appeared in the low quintile, and HR gradually increased as the quintile increased. Previously published cohort studies in AMI survivors reported that long-term exposure to PM_10_ was associated with an increased risk of heart failure and second MI [16]. In a cohort study of five European cities, the risk of hospital re-admissions due to cardiac causes was increased by daily concentrations of PM_10_, particle number concentrations (PNC), NO_2_, CO, and O_3_ in the MI population [19]. Many suggestive evidence have demonstrated that AP exposure is positively associated with cardiovascular disease incidence even in the general population [3, 6, 7, 20–25]. The results of this study was similar to other studies, wherein AP was associated with adverse health effects; however, there was a difference in pollutants that increased the risk. Particularly in the case of PM_2.5_, the data collection period was short; therefore, a relatively small number of subjects and short study periods were insufficient to determine an association with the clinical outcomes. Further research with sufficient study periods and number of subjects is needed to evaluate the association between PM_2.5_ and clinical outcomes. Moreover, in AP studies, characteristics according to exposure period, study population, location, and concentration were different for each study; therefore, it is difficult to obtain consistent results for every single pollutant [3, 26].

To reduce the risk of AP exposure, it is necessary not only to implement a government policy to reduce AP emissions but also to respond to individual exposure to high concentrations of AP. Wearing a face mask was shown to be an effective protection method to reduce the inhalation of PM, alleviate symptoms of cardiovascular disease, reduce blood pressure, and increase heart rate variability in patients with cardiovascular disease [27]. In addition, in a recent study, moderate to vigorous physical activity more than 5 times per week reduced the risk of cardiovascular disease and stroke even high PM_10_ concentration [28]. Education such as continuous reduction or prevention of AP exposure and encouraging indoor exercise when AP concentration is high, should be recommended for AMI patients and high-risk individuals.

This study had several limitations. First, it was likely that misclassification of patients to their exposure levels occurred because the addresses of patients were not available; thus patients who visited near or transferred hospitals could be misclassified. This study was conducted based on the concentration of ambient AP. The concentrations of exposure to each air pollutant at home or in the workplace were unavailable. Second, we were unable to detect significant adverse health associations with PM_2.5_, but this was likely due to the very limited sampling data available for this air pollution metric, which was monitored only in the last year of the study. Additional data and studies are needed to determine the association between PM_2.5_ and clinical events. Third, although the clinical factors influencing the prognosis were adjusted, factors such as socioeconomic status could not be adjusted due to the limitations of the study design. Fourth, all clinical results were confirmed by the data coordinators and researchers at each hospital. Although the main research center performed rigorous data management and training, input errors and misclassification of clinical events were possible because the sample size was large and many hospitals participated. Finally, the one-year follow-up period was relatively short to clarify the association with long-term AP exposure. Since the long-term prognosis is continuously poor in AMI patients compared with the general population, it is necessary to establish an association through studies with longer follow-up durations.

## Conclusion

In AMI patients, a higher concentration of SO_2_ and PM_10_, not only after but also before hospitalization, was associated with an increased risk of all-cause death. During the follow-up period, a higher concentration of SO_2_, CO, and PM_10_ was associated with an increased risk of any-revascularization up to 1-year using a nationwide prospective multicenter registry. We suggest that AMI patients and high-risk individuals establish a strategy to reduce or prevent exposure to high concentrations of AP.

## Supporting information

S1 Fig The cumulative incidence rate of all-cause death at different time points. (TIF)

S2 Fig Subgroup Analysis for adjusted hazard ratio and 95% confidence interval of the incidence of 30 days all-cause death according to increase 1 part per billion SO_2_ before symptom date. (TIF)

S3 Fig Subgroup Analysis for adjusted hazard ratio and 95% confidence interval of the incidence of 30 days all-cause death according to increase 0.1 part per million CO before symptom date. (TIF)

S4 Fig Subgroup Analysis for adjusted hazard ratio and 95% confidence interval of the incidence of 30 days all-cause death according to increase 1 part per billion O_3_ before symptom date. (TIF)

S5 Fig Subgroup Analysis for adjusted hazard ratio and 95% confidence interval of the incidence of 30 days all-cause death according to increase 1 part per billion NO_2_ before symptom date. (TIF)

S6 Fig Subgroup Analysis for adjusted hazard ratio and 95% confidence interval of the incidence of 30 days all-cause death according to increase 1 ㎍/㎥ PM_10_ before symptom date. (TIF)

S1 Table Distribution of annual average of air pollution concentration before symptom date. (DOCX)

S2 Table Association between annual average concentration before symptom date and all-cause death at different time points. (DOCX)

S3 Table Association between annual average concentration after symptom date and after 30 days to one-year all-cause death. (DOCX)
