# Supplementary figures and images for: Association of air pollution and 1-year clinical outcomes of patients with acute myocardial infarction
Source: PLoS One. 2022 Aug 1;17(8):e0272328. doi: 10.1371/journal.pone.0272328 (PMC9342741; doi:10.1371/journal.pone.0272328)

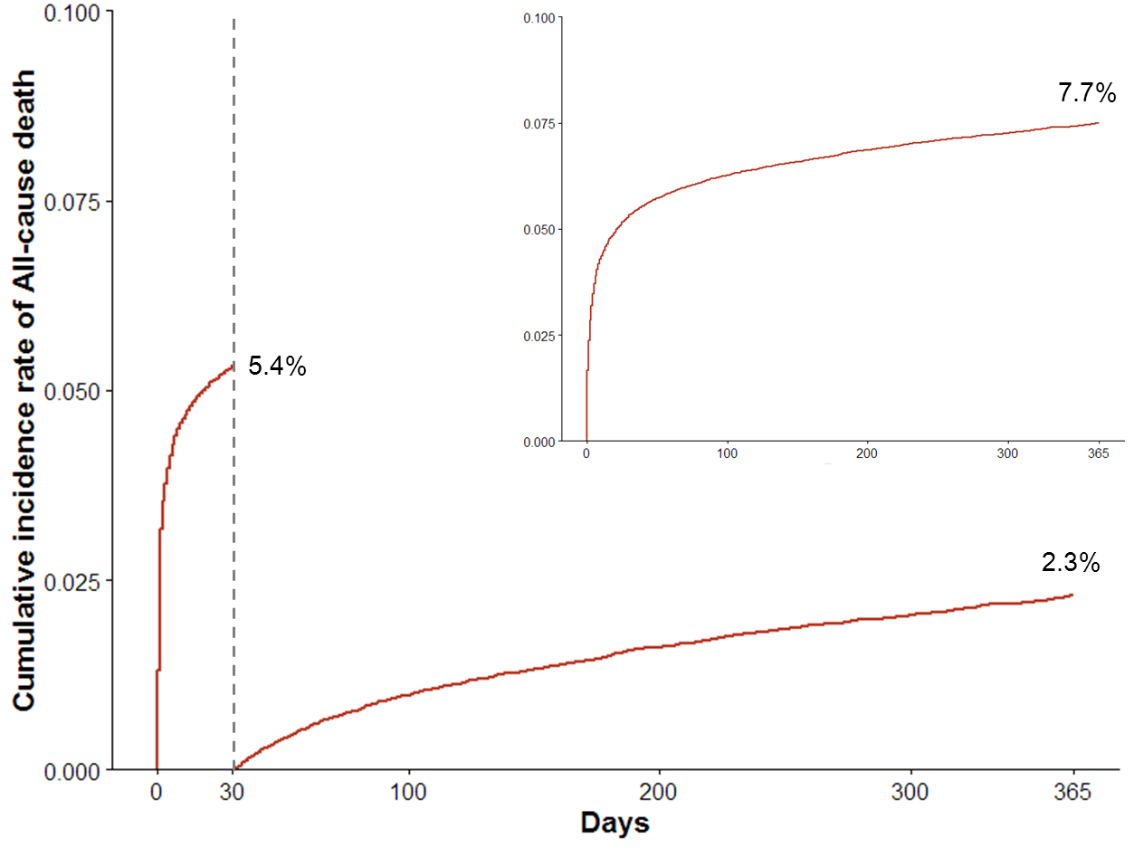

Supplement: S1 Fig — (TIF) [file pone.0272328.s001.tif]

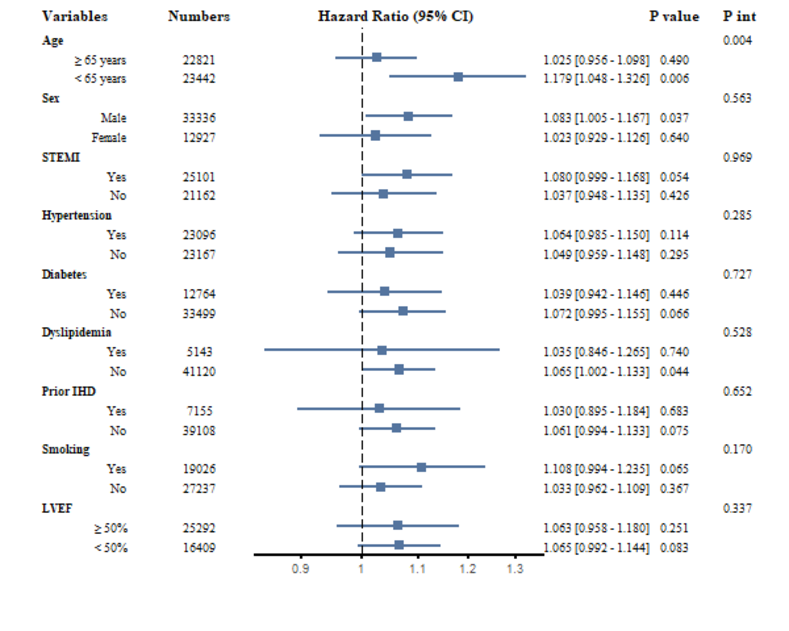

Supplement: S2 Fig — (TIF) [file pone.0272328.s002.tif]

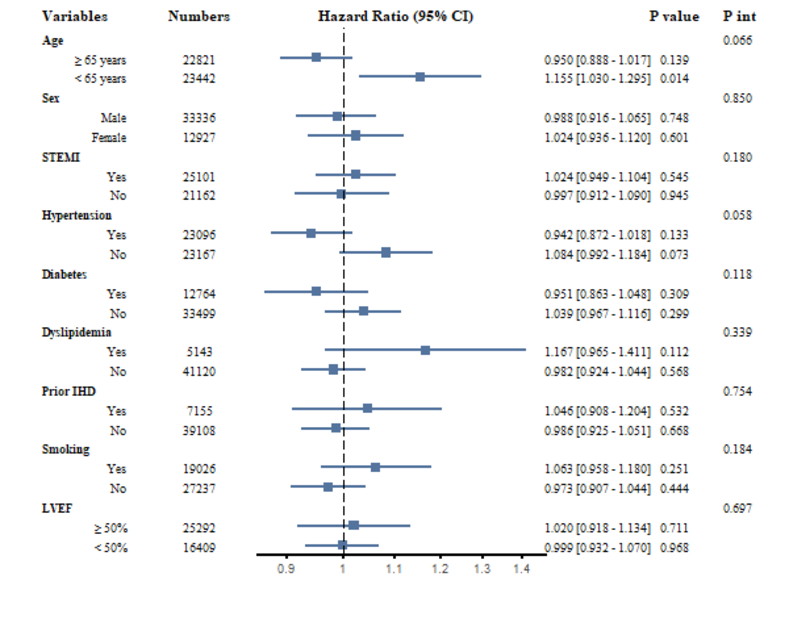

Supplement: S3 Fig — (TIF) [file pone.0272328.s003.tif]

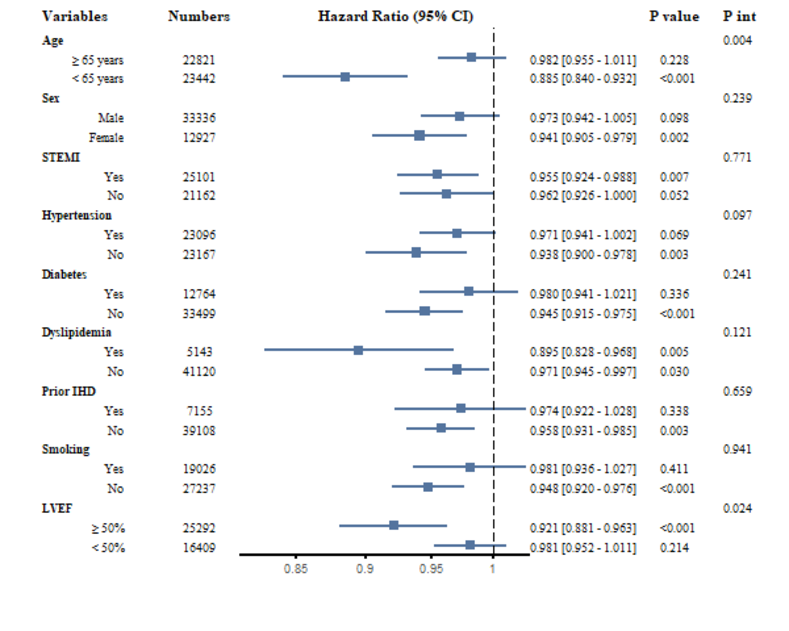

Supplement: S4 Fig — (TIF) [file pone.0272328.s004.tif]

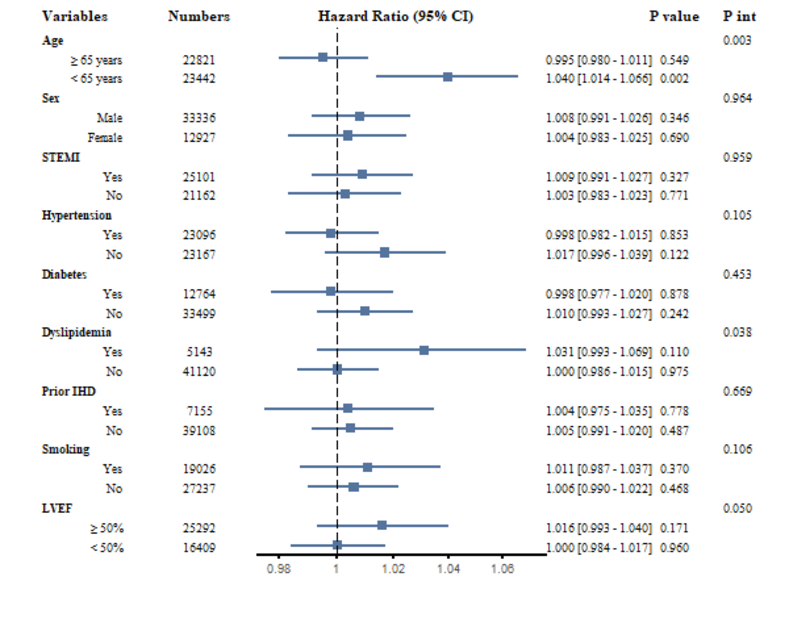

Supplement: S5 Fig — (TIF) [file pone.0272328.s005.tif]

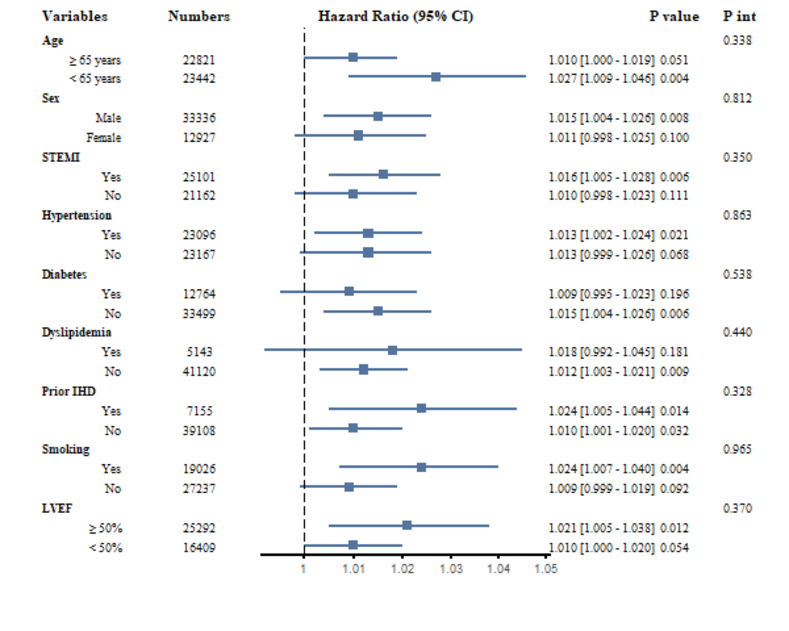

Supplement: S6 Fig — (TIF) [file pone.0272328.s006.tif]
