# Supplementary material for: Association of air pollution and 1-year clinical outcomes of patients with acute myocardial infarction
Source: PLoS One. 2022 Aug 1;17(8):e0272328. doi: 10.1371/journal.pone.0272328 (PMC9342741; doi:10.1371/journal.pone.0272328)
Supplement: S1 Table — (DOCX) [file pone.0272328.s007.docx]

S1 Table. Distribution of annual average of air pollution concentration before symptom date

|  | **SO_2_, ppm** | **CO, ppm** | **O_3_, ppm** | **NO_2_, ppm** | **PM_10_, ㎍/㎥** |
| --- | --- | --- | --- | --- | --- |
| 0 - 20% (Q1) | 0.0014 - 0.0040 | 0.2028 - 0.5031 | 0.0037 - 0.0169 | 0.0070 - 0.0210 | 18.25 - 43.20 |
| 20 - 40% (Q2) | 0.0040 - 0.0046 | 0.5031 - 0.5785 | 0.0169 - 0.0201 | 0.0210 - 0.0242 | 43.20 - 48.59 |
| 40 - 60% (Q3) | 0.0046 - 0.0054 | 0.5785 - 0.6457 | 0.0201 - 0.0224 | 0.0242 - 0.0287 | 48.59 - 53.17 |
| 60 - 80% (Q4) | 0.0054 - 0.0064 | 0.6457 - 0.7383 | 0.0224 - 0.0253 | 0.0287 - 0.0357 | 53.17 - 60.95 |
| 80 - 100% (Q5) | 0.0064 - 0.0136 | 0.7383 - 1.4976 | 0.0253 - 0.0437 | 0.0357 - 0.0812 | 60.95 - 99.14 |
| Median | 0.0049 | 0.6076 | 0.0212 | 0.0258 | 50.43 |
| Mean | 0.0053 | 0.6268 | 0.0210 | 0.0284 | 52.47 |
| Interquartile range (IQR) | 0.0020 | 0.1889 | 0.0068 | 0.0124 | 13.99 |
