# Supplementary material for: Association of air pollution and 1-year clinical outcomes of patients with acute myocardial infarction
Source: PLoS One. 2022 Aug 1;17(8):e0272328. doi: 10.1371/journal.pone.0272328 (PMC9342741; doi:10.1371/journal.pone.0272328)
Supplement: S2 Table — (DOCX) [file pone.0272328.s008.docx]

S2 Table. Association between annual average concentration before symptom date and all-cause death at different time points.

|  | **All-cause death up to 30-day** | | **All-cause death after 30-day to one year** | |
| --- | --- | --- | --- | --- |
|  | **Hazard Ratio (95% CI)** | **P-value** | **Hazard Ratio (95% CI)** | **P-value** |
| SO_2_, ppb | 1.084 (1.016 - 1.157) | 0.015 | 1.008 (0.931 - 1.092) | 0.844 |
| SO_2_ Q_1_ | **Reference** |  | **Reference** |  |
| SO_2_ Q_2_ | 0.924 (0.737 - 1.159) | 0.494 | 1.041 (0.843 - 1.285) | 0.711 |
| SO_2_ Q_3_ | 0.971 (0.771 - 1.224) | 0.805 | 0.932 (0.738 - 1.176) | 0.553 |
| SO_2_ Q_4_ | 1.539 (1.186 - 1.996) | 0.001 | 0.978 (0.729 - 1.312) | 0.882 |
| SO_2_ Q_5_ | 1.560 (1.164 - 2.092) | 0.003 | 1.084 (0.770 - 1.527) | 0.644 |
| CO, 0.1 ppm | 0.979 (0.919 - 1.043) | 0.515 | 1.020 (0.937 - 1.110) | 0.650 |
| CO Q_1_ | **Reference** |  | **Reference** |  |
| CO Q_2_ | 1.080 (0.835 - 1.397) | 0.558 | 1.131 (0.889 - 1.439) | 0.316 |
| CO Q_3_ | 0.958 (0.727 - 1.262) | 0.762 | 0.973 (0.740 - 1.280) | 0.844 |
| CO Q_4_ | 1.154 (0.870 - 1.530) | 0.321 | 1.023 (0.756 - 1.384) | 0.885 |
| CO Q_5_ | 1.066 (0.767 - 1.481) | 0.705 | 0.784 (0.535 - 1.150) | 0.214 |
| O_3_, ppb | 0.960 (0.933 - 0.987) | 0.004 | 0.977 (0.943 - 1.012) | 0.200 |
| O_3_ Q_1_ | **Reference** |  | **Reference** |  |
| O_3_ Q_2_ | 0.604 (0.472 - 0.772) | <0.001 | 1.522 (1.065 - 2.175) | 0.021 |
| O_3_ Q_3_ | 0.511 (0.380 - 0.686) | <0.001 | 1.485 (0.972 - 2.268) | 0.068 |
| O_3_ Q_4_ | 0.586 (0.425 - 0.807) | 0.001 | 1.216 (0.779 - 1.900) | 0.389 |
| O_3_ Q_5_ | 0.644 (0.448 - 0.926) | 0.018 | 0.942 (0.576 - 1.542) | 0.813 |
| NO_2_, ppb | 1.000 (0.985 - 1.015) | 0.993 | 0.981 (0.962 - 1.000) | 0.054 |
| NO_2_ Q_1_ | **Reference** |  | **Reference** |  |
| NO_2_ Q_2_ | 0.733 (0.571 - 0.940) | 0.014 | 1.061 (0.830 - 1.356) | 0.638 |
| NO_2_ Q_3_ | 0.848 (0.642 - 1.121) | 0.247 | 0.972 (0.721 - 1.311) | 0.851 |
| NO_2_ Q_4_ | 0.795 (0.520 - 1.214) | 0.288 | 1.056 (0.673 - 1.658) | 0.813 |
| NO_2_ Q_5_ | 0.991 (0.610 - 1.611) | 0.970 | 0.654 (0.378 - 1.133) | 0.130 |
| PM_10_, ㎍/㎥ | 1.011 (1.002 - 1.021) | 0.020 | 0.998 (0.987 - 1.009) | 0.664 |
| PM_10_ Q_1_ | **Reference** |  | **Reference** |  |
| PM_10_ Q_2_ | 0.885 (0.707 - 1.108) | 0.285 | 1.242 (0.985 - 1.565) | 0.067 |
| PM_10_ Q_3_ | 1.014 (0.798 - 1.289) | 0.907 | 1.124 (0.879 - 1.438) | 0.351 |
| PM_10_ Q_4_ | 1.465 (1.112 - 1.928) | 0.007 | 1.015 (0.732 - 1.408) | 0.928 |
| PM_10_ Q_5_ | 1.804 (1.310 - 2.484) | <0.001 | 0.718 (0.470 - 1.098) | 0.126 |
| Adjusted by Age, Sex, Body mass index, Smoker, ST-segment elevation myocardial infarction, Hypertension, Diabetes mellitus, Dyslipidemia, Stroke, Heart failure, Previous ischemic heart disease, Percutaneous coronary intervention, multi-vessel disease, Left Main Disease, Cardiopulmonary resuscitation, Left ventricular ejection fraction and symptom date  ppm; part per million, ppb; part per billion | | | | |
