# Supplementary material for: Association of air pollution and 1-year clinical outcomes of patients with acute myocardial infarction
Source: PLoS One. 2022 Aug 1;17(8):e0272328. doi: 10.1371/journal.pone.0272328 (PMC9342741; doi:10.1371/journal.pone.0272328)
Supplement: S3 Table — (DOCX) [file pone.0272328.s009.docx]

S3 Table. Association between annual average concentration after symptom date and after 30 days to one-year all-cause death.

|  | **Hazard Ratio (95% CI)** | **P-value** |
| --- | --- | --- |
| SO_2_, ppb | 1.084 (1.003 - 1.172) | 0.042 |
| SO_2_ Q_1_ | **Reference** |  |
| SO_2_ Q_2_ | 1.260 (1.010 - 1.573) | 0.041 |
| SO_2_ Q_3_ | 1.165 (0.933 - 1.455) | 0.178 |
| SO_2_ Q_4_ | 1.078 (0.802 - 1.449) | 0.619 |
| SO_2_ Q_5_ | 1.334 (0.951 - 1.872) | 0.095 |
| CO, 0.1 ppm | 1.034 (0.941 - 1.136) | 0.484 |
| CO Q_1_ | **Reference** |  |
| CO Q_2_ | 1.353 (1.039 - 1.760) | 0.025 |
| CO Q_3_ | 1.326 (0.987 - 1.783) | 0.061 |
| CO Q_4_ | 1.175 (0.854 - 1.618) | 0.323 |
| CO Q_5_ | 0.768 (0.519 - 1.137) | 0.188 |
| O_3_, ppb | 0.988 (0.957 - 1.020) | 0.462 |
| O_3_ Q_1_ | **Reference** |  |
| O_3_ Q_2_ | 1.366 (0.958 - 1.946) | 0.085 |
| O_3_ Q_3_ | 1.280 (0.840 - 1.949) | 0.251 |
| O_3_ Q_4_ | 1.374 (0.890 - 2.121) | 0.152 |
| O_3_ Q_5_ | 1.050 (0.653 - 1.690) | 0.841 |
| NO_2_, ppb | 0.997 (0.977 - 1.017) | 0.763 |
| NO_2_ Q_1_ | **Reference** |  |
| NO_2_ Q_2_ | 0.951 (0.743 - 1.217) | 0.690 |
| NO_2_ Q_3_ | 1.285 (0.957 - 1.725) | 0.095 |
| NO_2_ Q_4_ | 0.991 (0.665 - 1.477) | 0.965 |
| NO_2_ Q_5_ | 1.299 (0.805 - 2.095) | 0.284 |
| PM_10_, ㎍/㎥ | 1.021 (1.009 - 1.033) | <0.001 |
| PM_10_ Q_1_ | **Reference** |  |
| PM_10_ Q_2_ | 1.152 (0.876 - 1.514) | 0.313 |
| PM_10_ Q_3_ | 1.723 (1.344 - 2.208) | <0.001 |
| PM_10_ Q_4_ | 1.145 (0.802 - 1.635) | 0.456 |
| PM_10_ Q_5_ | 1.579 (1.034 - 2.411) | 0.034 |
| PM_2.5_, ㎍/㎥ | 1.200 (0.787 - 1.830) | 0.398 |
| PM_2.5_ Q_1_ | **Reference** |  |
| PM_2.5_ Q_2_ | 0.736 (0.306 - 1.768) | 0.493 |
| PM_2.5_ Q_3_ | 1.155 (0.134 - 9.930) | 0.895 |
| PM_2.5_ Q_4_ | 1.758 (0.099 - 31.316) | 0.701 |
| PM_2.5_ Q_5_ | 0.784 (0.020 - 31.463) | 0.897 |
| Adjusted by Age, Sex, Body mass index, Smoker, ST-segment elevation myocardial infarction, Hypertension, Diabetes mellitus, Dyslipidemia, Stroke, Heart failure, Previous ischemic heart disease, Percutaneous coronary intervention, Multi-vessel disease, Left Main Disease, Cardiopulmonary resuscitation, Left ventricular ejection fraction and symptom date  ppm; part per million, ppb; part per billion | | |
